# Lardner’s Natural Philosophy, Etc.

**Published:** 1854-05

**Authors:** 


*Lardner's Natural Philosophy, Course of Astronomy and Meteorology. Lea & Blanchard, Philadelphia,* 1854. (*Received from D. B. Cooke & Co., Chicago.*)

THIS is the third and last of a valuable series of "Handbooks" on a subject ever interesting to the scientific physician. Although only collateral to the science of medicine, the subject of Meteorology which commences this volume presents strong claims to those who would read the great book of nature aright. Most of the operations of nature's harmonic institutions depend upon climatic and meteorological causes, almost inappreciable in a state of health, but sorrowfully apparent when the victim of disease.

The true nature of disease must remain hidden until we can discover "what is life"—not as an abstract conception, but a palpable entity; yet statistical science can advance our knowledge of the relation between physical laws, and those which govern the more subtle essence. Writers have ascribed many diseases, especially those of an epidemic character, to peculiar conditions of the atmosphere—electrical and hygrometrical, as acting *per se*, or giving intensity to predisposing conditions, or more efficient causes. Dr. Prout found the air in London just before the cholera broke out there, in 1832, much heavier than usual. We consider that no physician can be an *observer* of disease, or advance its etiology, without knowledge of the principles of meteorology.

The work before us professes to bring the study "up to the day;" it cannot however, rank with other treatises on the same subject, indeed we think it far inferior to an indigenous work, by Brocklesly, Professor of Natural Philosophy in Trinity College, Hartford. The more voluminous and elaborate treatises on this subject are, *Kœmptz' complete course of Meteorology*, *Muller's Physics* and *Meteorology*, and the paper in the *Annales de chemie et de Physique*, by Regnault and Pouillet.

The work begins with the subject of Terrestrial Heat. It is well known that the temperature of the earth decreases in going from the equator to the poles, but this decrease is not equal for the same latitude, but varies many degrees. This fact led Humboldt to establish his system of *isothermal lines*, or lines passing around the earth through places having the same temperature.—The decrease in temperature from the equator would be in direct ratio with our advance towards the polar seas, were there not disturbing causes—as difference in geological formation, presenting surfaces having unequal powers of absorption and radiation; the proximity of large bodies of salt or fresh water, mountain chains, primeval forests, littoral curvatures, configuration of coast, etc. Climate may vary on the same isothermal line, from the difference in vegetable productions; London, Pekin, New York are nearly on the same isothermal line, but their climates are extremely different. Climate is modified by elevation above the sea level; the thermometer falling one degree for every three hundred and twenty feet of altitude. After attaining an elevation of 15,730 feet in latitude 0°10′ N. we find perpetual snow, and a mean annual temperature of 34°.

In connection with this subject, the author has not entered fully into the cause of variation of climate:—nor has he attempted to explain the interesting fact of the lower temperature of the western continent, which is liable to more sudden and greater changes than the eastern, and the superior warmth of the western part of our own continent. We will assist him. On the eastern continent we find the Carpathian mountains in Austria; the Balkan in European Turkey; the Alps in Switzerland, the Appenines in Italy; the Pyrenees and the ranges ef the Sierre Morena, and Sierra Nevada, in Spain, and in India the lofty ranges of the Grants, and Himalaya protecting the ultramontane countries from hyperborean winds. The borders of the Black Sea are colder than the neighboring countries, because the polar winds sweep uninterruptedly through the opening between the Caucasus and the mountains of Transylvania. On the western continent we find no barriers presented to the polar winds, they sweep from the frozen ocean to the Gulf of Mexico; they strike against the Rocky Mountaïns obliquely and become a N. W. wind, bringing cold and storms to the great interior valley. It is the alternation of polar and equatorial winds which causes the extremes of heat and cold, and gives a proclivity to such centripetal diseases, as Influenza, and pulmonary affections in the winter, and vernal months, and Colic, Cholera morbus, and its congeners in the hot season. In the middle latitudes of the Pacific the prevalent winds are from the S. W., these carry their mild oceanic temperature to the western coast of America, and produce that mild and equable climate, peculiar to the countries contained between the range of the Rocky Mountains in the north, the Cordilleras in the middle, and the Andes in the southern divisions, and the sea coast.

In chap. 111, we have the subject of hygrometry, together with a description of Daniell's, Saussure's, Le Roy's, and Regnault's hygrometry, and August's psychrometer. We consider that of Regnault the most delicate and reliable; but its expensiveness will preclude its general use. We sincerely hope to see this subject taken up by industrious observers: it presents a rich field for scientific investigation. The relation of the hygrometric condition of the atmosphere to our vernal and autumnal fevers is, we believe, more intimate than superficial observers imagine. We have, ourselves, noticed that the intensity of cholera, and malarious fevers, has always coinceded with a high "*dew point*." In the Mediteranean the pestilential Siroc comes loaded with moisture. Did our limits admit we would wish to take enter interesting details, in connection with the subject.

The subject of Hoar frost is very meagrely treated in sec. 2249. It has been observed that hoar frost forms first in valleys: this is due to the upward radiation of terrestrial heat, from the protection afforded by the surrounding hills. Our author has omitted this explanation.

The effects of atmospheric electricity on animal and vegetable matter are obscure, but not unobservable. There are but few of nervous–sanguine temperament who have not felt an indescribable sense of oppression and anxiety, of restlessness, and debility of the muscular system, immediately before a thunder storm—evidencing some palpable disturbance of the electricity of the body, by this subtle agent in the atmosphere. Its effects, too, on vegetable matter, are scarcely less mysterious. In the department of the Cote d'Or, Saone, and Loire in France, where the fine wines of Burgundy are produced, the fermentation of a whole vintage has been disturbed by a thunder storm. It would seem from this that electricity disturbs the production of carbonic acid, which Pouillet has observed to escape in a negatively electrified state.

While this book contains much valuable information for a student, it does not fulfill all it promises. Many of the subjects are passed over too hastily and some valuable matter, in an elementary work, wholly omitted. The style of Dr. Lardner is never attractive and this book forms no exception to the general rule.

W. H. T.

---

From and after the issue of this No. of the North Western Medical and Surgical Journal, Dr. N S. Davis will take the place of the undersigned as one of its editors.

The well deserved and high reputation of Dr. Davis as a writer and teacher will be a sufficient guarantee to its subscribers, that hereafter, it will continue to merit what it has always had and is now receiving, a liberal support from the Physicians of the North West.

